# Development of an individual helmet orthosis for infants based on a 3D scan

**DOI:** 10.1186/s41205-023-00187-7

**Published:** 2023-08-16

**Authors:** Fabian Kropla, Martin Hoffmann, Dirk Winkler, Matthias Krause, Sebastian Scholz, Ronny Grunert

**Affiliations:** 1 Department of Neurosurgery, University of Leipzig Medical Center, Liebigstr. 20, 04103 Leipzig, Germany; 2 Department for Mechanical Engineering, Chemnitz University of Technology, 09111 Chemnitz, SN Germany; 3 Fraunhofer Institute for Machine Tools and Forming Technology, 02763 Zittau, SN Germany

## Abstract

An early childhood skull deformity can have long-term health and aesthetic consequences for the growing toddler. Individual helmet therapy aims at a healthy growth of the skull shape, although not every helmet shape guarantees an optimal result. To ensure an optimal fit, a scanning procedure based on a hand-held surface scanner was evaluated.

The new helmet orthosis has an inner layer adapted to the shape of the head, which can be exchanged depending on the growth stage without changing the outer layer.

In collaboration with surgeons and engineers, a new helmet orthosis concept was developed that is intended to offer improvements in wearing comfort, overall weight, fit and user-friendliness compared to conventional systems. In the course of the development process and in constant exchange with parents, a multi-layer helmet system with generous perforations was created using additive manufacturing processes. The new helmet shape promises easier handling, especially through the closure system.

The helmet shape developed in this study is of high quality, especially in terms of fitting accuracy. Unpleasant perspiration is significantly reduced. The integration of the closure as a direct component of the helmet represents a secure closure option.

## Introduction

**Fig. 1:**
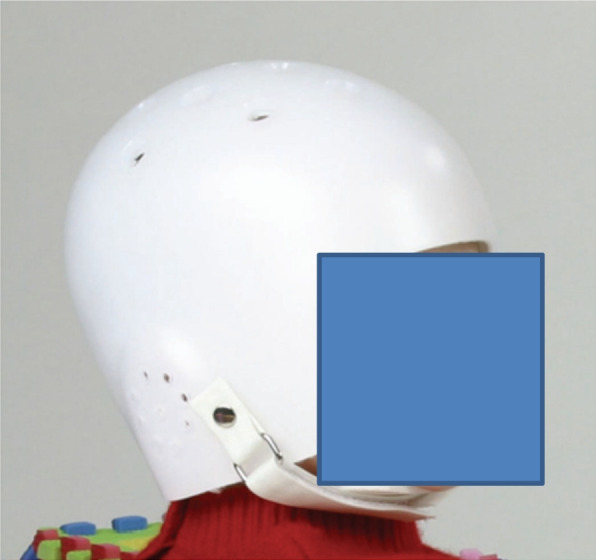
The first helmet orthosis developed by S.K.Clarren in 1979. [8]

Deformity in children's head shape has various etiologies such as prenatal constriction in the uterus, perinatally due to trauma, such as the breech presentation, during birth, as well as postnatal functional or anatomical constraints. A permanent supine position of the small child can also lead to undesirable deformities [3, 16]. In the course of a US-American "Back to Sleep" campaign, in which parents were encouraged to put their children on their backs while sleeping, the rate of sudden infant deaths dropped considerably. However, this also led to an increase in the number of cranial deformities in children caused by lying on their heads for long periods of time, which is why the development of head disorders became increasingly important. As early as 1979, "We hypothesized that if the pressure of a rapidly growing brain against a flat surface would flatten the skull, then pressure against a concave surface should round it back again. We have found that individualized plastic helmets could remold the rhomboid-shaped head into a more usual form" [6], S. 43). Following this discovery, the first helmet orthoses for infants were developed. This hasproven to be a successful therapy and now represents the therapeutic standard for positional plagiocephalus. Figure 1 shows a conventional children's helmet based on the so-called Clarren design. This helmet consists of a large plastic scale, a fastening buckle that is tightened around the chin and a few ventilation holes. In the course of time, the shape of the helmet was optimised, especially with regard to wearing comfort and ventilation.

The fitting of the orthosis must always be patient-specific, as the deformity is highly individual. Skull deformities can basically be divided into two clinical pictures, plagiocephalus and brachycephalus. Plagiocephalus describes an asymmetrical shift in the length–width relationship of the head, whereas brachycephalus describes the compression of one side of the head. Helmet therapy is primarily used for deformities of the plagiocephalus.

**Fig. 2:**
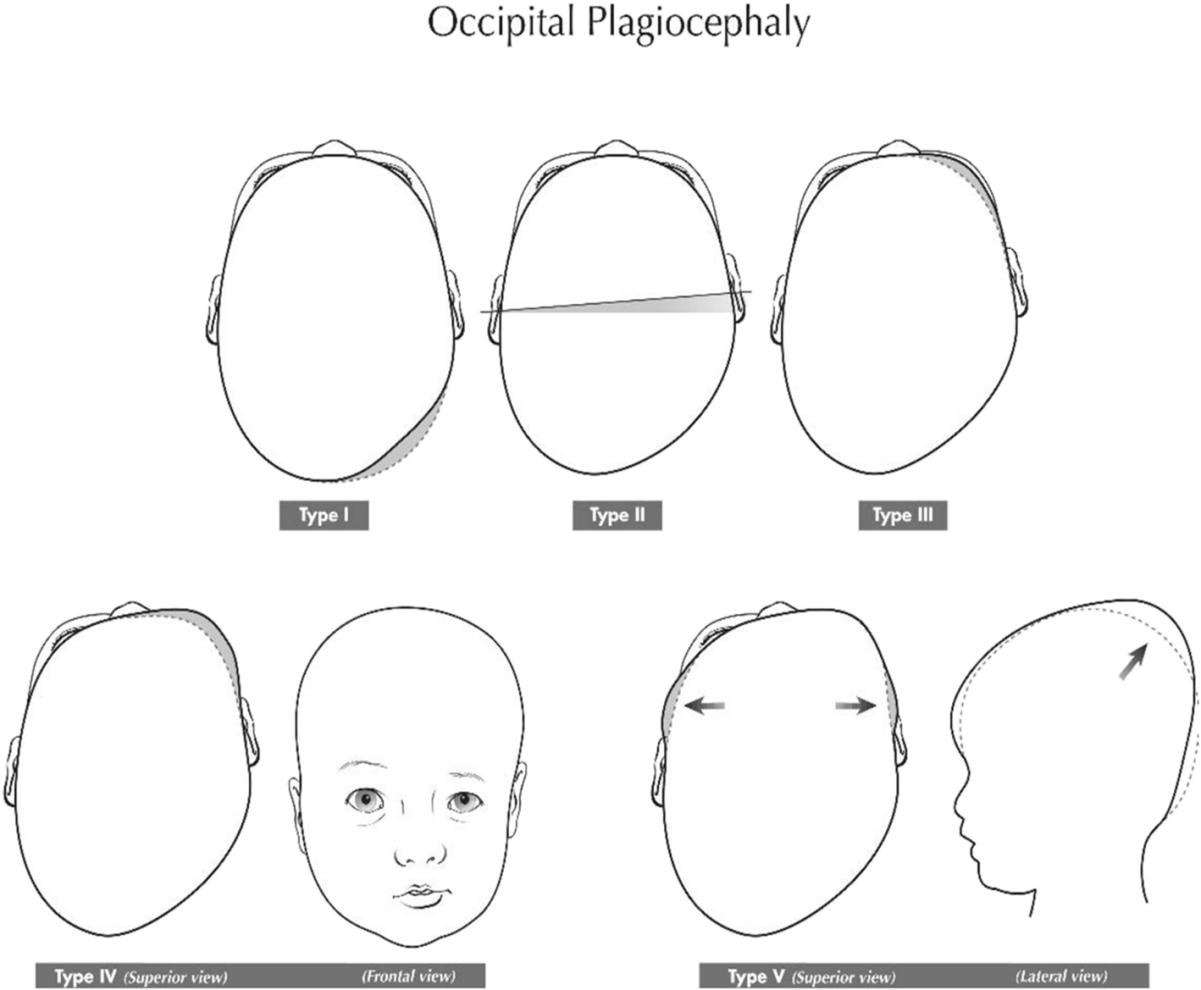
Classification of plagiocephaly according to [2]

The development of a plagiocephaly can be subdivided into five phases [2] as illustrated in Fig. 2. Depending on the degree of severity, type I describes a mild deformation with only one-sided flattening of the occiput. The subsequent types show an increasing deformation of the skull. Type V is defined as an extreme case, which is defined by additional ear displacement, facial involvement and laterally and apically projecting skull.

**Fig. 3:**
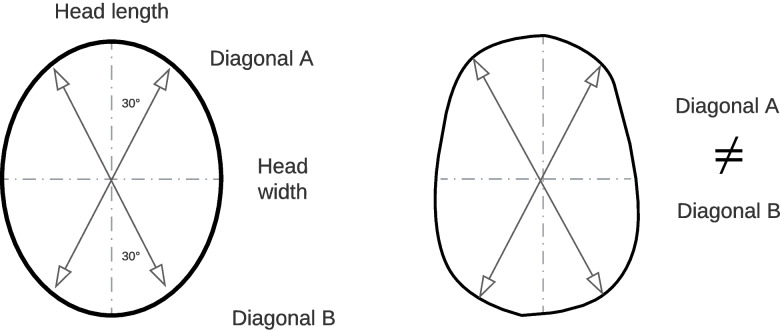
Illustration of the CVA in a healthy and a deformed skull

Various clinical parameters are used to determine the severity of a cranial deformity. In addition to circumference and head diameter, the diagonal difference, the so-called Cranial Vault Ansymmetry (CVA), is important. The CVA is the difference between the two diagonal values, which are at an angle of 30° to the middle cranial axis (skull longitudinal axis) (Fig. 3). For example, asymmetries with a diagonal difference of less than 10 mm are usually not visible in adulthood and therefore do not need to be treated in infancy.

To give a numerical value to the cranial curvature, the Cranial Vault Asymmetry Index (CVAI) is used. This value indicates a percentage value relative to the skull, which can be calculated using the following formula. A CVAI of 12% or more is a severe deformity with absolute indication for treatment.

$$CVAI=\frac{Diagonal\;A-Diagonal\;B}{Diagonal\;B}\times100\%$$

Slight asymmetries, in the range between 3 mm and a CVAI of 1.5% to 3.1%, are considered normal values in the literature and do not need to be treated [14].

Another indicator of skull deformity is the ear offset, which is determined numerically by an imaginary line between the auricles and the longitudinal axis of the skull [10].

Head asymmetries should be treated as soon as possible after diagnosis, as they are not only detrimental to aesthetics. In addition, facial asymmetries as well as jaw and tooth position problems can occur in the course of life. Furthermore, the asymmetric cranial weight can cause extreme muscular imbalances of the neck muscles, which can cause shoulder, neck and head pain after verticalisation [16]. The retroperspective study by [15] points out various complications of helmet therapy. For example, with a frequency of 10.9%, pressure sores can occur due to excessive contact and pressure on the skin. Furthermore, skin reddening can occur in response to the alcohol used to clean the helmet or skin infections due to insufficient cleaning of the helmet. Occasionally, bacterial abscesses were also described, which were caused by the helmet not being fitted and worn despite pressure points. A poor fit of the helmet, a lack of therapeutic success and non-compliance are further complications in connection with helmet therapies.

## Material and methods

### 3D-Scan

**Fig. 4:**
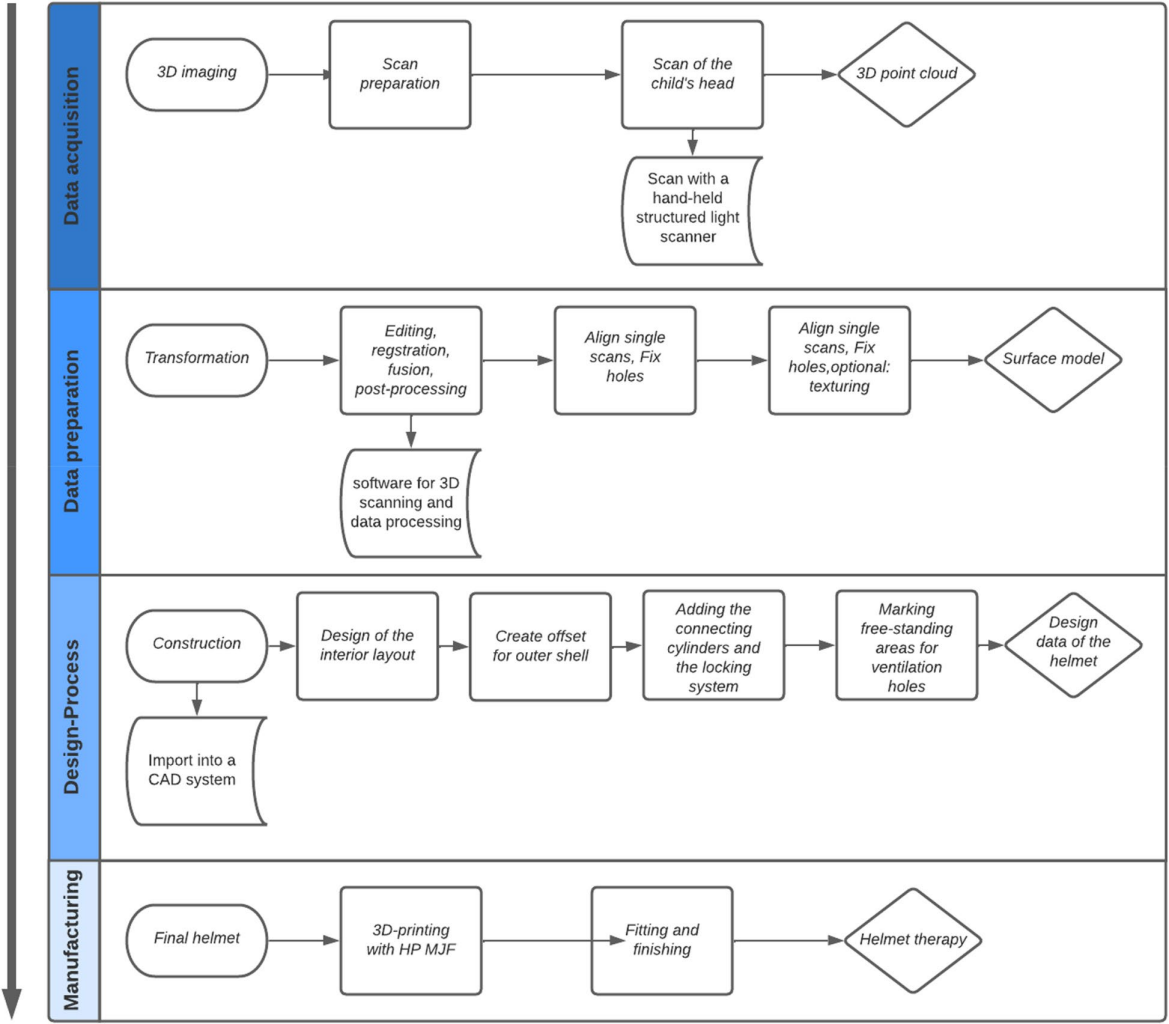
Overall process for the individual manufacture of a helmet orthosis

The basis for the design process is an individual 3D scan. In the selection process, a choice was made between a laser triangulation scanner and a structured light scanner. The 3D scan of the head is then to be used as a watertight 3D model in further design programmes Fig. 4.

Since 3D scanning technology has already proven itself in orthotic development [5, 7, 9, 12, 13]. As 3D scanning technology has proven itself in the production of children's helmets, it is also used for the product development of this helmet, whereby the accuracy of the scanning process plays a major role. 3D scanning technology is a non-contact digitisation process that does not emit dangerous radiation compared to a CT scan. It is also a convenient alternative to a plaster cast. The scanning system should have the highest possible scanning speed, which contributes to low motion sensitivity. This is very important because the patients are small children who cannot sit still constantly during the scanning process. The possible scanning volume should also be suitable for the size of the heads and, if necessary, scan surrounding objects as well, to always have enough orientation points to minimise the risk of the scanning process being interrupted. In addition, user-friendliness plays a major role, which is why the scanner should at best be wireless and mobile.

**Fig. 5:**
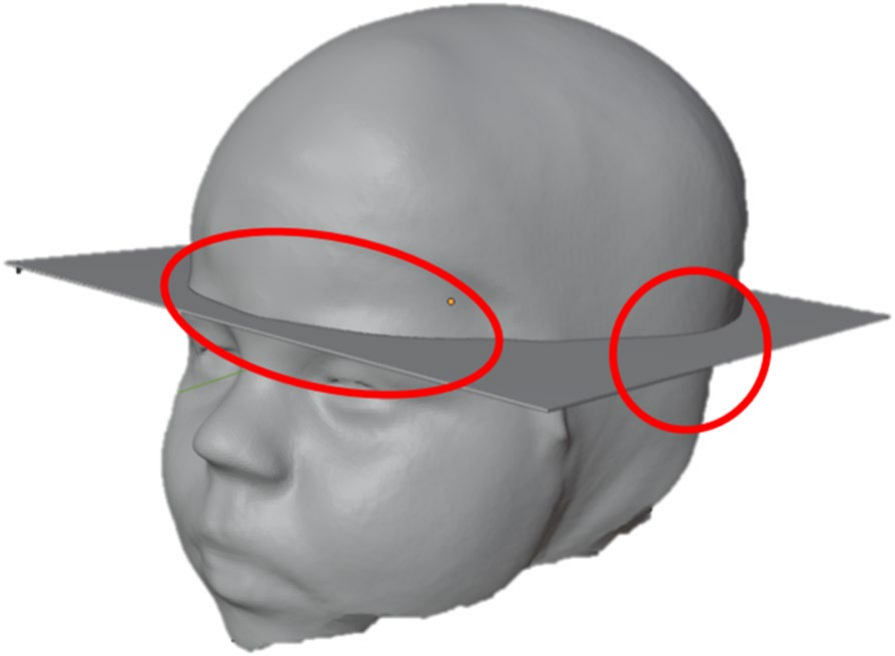
Relevant head regions Arcus superciliearis and Helix auriculae for measuring the head

Despite the increasing accuracy of surface scanners, there is still a risk that the scan, which is the later basis for the 3D model and thus the orthosis, achieves an insufficient result. Therefore, it is all the more important to carry out a quantitative check of the measured values obtained from the scan and to discuss them. As part of a consultation for cranial deformities at Leipzig University Hospital and the Clinic and Polyclinic for Neurosurgery, 15 infants who are no more than twelve months old are used as test subjects. For the comparison, the head is measured on the one hand with an analogue measuring tape, the seca 201 circumference measuring tape, and on the other hand with a 3D modelling programme for the scanned variants. The measurement is carried out on a significant anatomical structure. For this purpose, the head circumference is measured along the arcus superciliearis (Fig. 5) above the eye socket and above the helix auriculae above the auricle.

### Design

The helmet orthosis uses the growth of the head to correct its shape. The helmet rests on the sensitive areas of the head. The aim is to suppress growth in these areas and at the same time to allow shape-guided growth into the ideal shape in the flattened areas.

The basis for the constructive development step is a utility value analysis with the following evaluation criteria: stability, weight, protruding components, prevention of manipulation by patients, operability, possibility of repair, manufacturing costs and aesthetics. The weighting of the decision factors is done according to the pair comparison method. Two criteria are compared and it is decided which of these factors is more relevant.

Within the framework of this project, the focus was on the development of an individual helmet that always adapts to the shape of the head during the course of therapy. This is achieved by a modular helmet design in which the inner layer, which rests on the head, can be replaced while the outer layer is retained. In order to connect the two layers, a push-button system was developed that allows the inner layer to be replaced quickly and without tools.

**Fig. 6:**
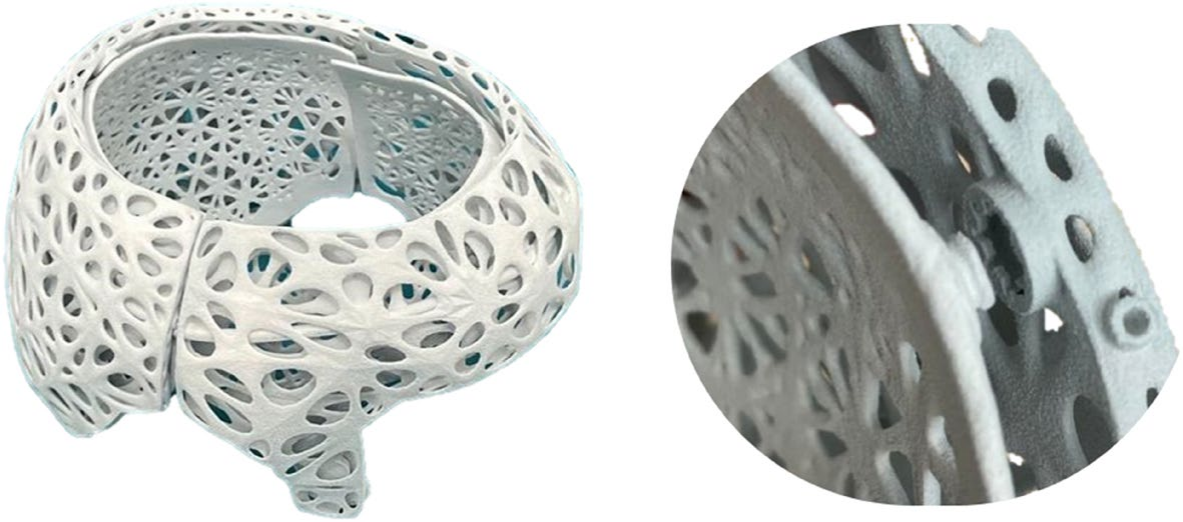
First prototype with push-button closure system

Figure 6 shows the helmet in an early prototype state. To improve ventilation, special perforations have been made in both helmet layers to prevent excessive perspiration.

### Manufacturing with 3D Printing

Powder Bed Fusion (Multi Jet FusionHewlett-Packard, California, USA) is based on a powdered starting material that is melted by heat-conducting liquids called agents and an infrared light [1]. This hardware platform is included in cleared patthways for patient care in the United States [11]. and Power Bed Fusion is used in Europe [4]. For this clinical application, it is particularly important that the complex component structures have an almost isotropic material behaviour; this is an advantage for the uneven loading of the helmet. In addition, the Nylon PA12 material used for this project is biocompatible, and it can be sterilised if necessary.

## Results

### 3D-Scan

The advantage of the structured light scanner is that it can be used safely on children. Laser triangulation scanners can pose a risk to the cornea if there is direct eye contact. Since it cannot be ensured that children in particular do not look into the light source, a structured light scanner is used. Other systems such as photgrammetry and time-of-flight technologies are out of the question because their accuracy and detection speed are too low.

For the digitisation of the children's heads, the 3D scanner ArtecLeo was used, which promises an accuracy of 0.1 mm and a 3D reconstruction rate of 80 FPS. In addition, it is a hand-held 3D scanner, which facilitates handling, especially a 360° scan.

The scanning methodology was validated on 15 subjects at Leipzig University Hospital. Eleven boys and four girls aged between six and twelve months served as test subjects (Table 1). The digitally and analogue braked children's heads did not all show plagiocephaly. Three analogue and three digital measurements were taken for each child, resulting in the mean value.

**Table 1:** Validation of the scanning methodology with 15 subjects

			Scan				Measuring tape			
			Head circumference in cm				Head circumference in cm			
Child	Gender	Age in months	x_1_	x_2_	x_3_	${\overline{X} }_{\mathrm{s}}$	x_1_	x_2_	x_3_	${\overline{X} }_{\mathrm{m}}$
1	m	6	43,9	44,0	44,2	44,03	43,9	44,2	43,7	43,93
2	m	8	45,6	45,4	45,9	45,63	45,4	45,5	45,3	45,40
3	f	6	42,1	42	41,9	42,00	41,5	41,9	41,6	41,67
4	m	5	43,3	43	42,8	43,03	43,2	42,5	42,9	42,87
5	f	7	43,3	43,2	43,0	43,17	43,2	43,0	42,9	43,03
6	m	10	46,3	46,1	46,0	46,13	46	45,8	46,2	46,00
7	m	5	42,1	41,8	40,7	41,53	41,0	41,7	40,5	41,07
8	m	9	45,7	45,5	45,5	45,57	45,3	45,4	45,3	45,33
9	m	7	44,5	44,7	44,5	44,57	44,3	44,4	44,6	44,43
10	f	8	43,9	44,2	44,1	44,07	43,8	43,9	43,8	43,83
11	m	11	46,8	46,9	47	46,90	46,7	46,8	46,7	46,73
12	m	6	44,5	44,3	44,3	44,37	44,2	43,7	44,1	44,00
13	f	5	42,1	41,8	41,3	41,73	41,5	41,8	40,7	41,33
14	m	8	45,4	45,2	45,0	45,20	44,9	45,3	45,1	45,10
15	m	7	44,2	44,3	44,6	44,37	44,1	44,3	44,2	44,20

Table 2 shows the difference between the scanned and analogue measured values, the maximum standard deviation and the mean value of the standard deviation.

**Table 2:** Difference of the determined mean values as well as average and maximum standard deviation

Child	${\overline{X} }_{\mathrm{s}}-{\overline{X} }_{\mathrm{m}}$	σ_s_	σ_m_
1	0,10	0,12	0,21
2	0,23	0,21	0,08
3	0,33	0,08	0,17
4	0,17	0,21	0,29
5	0,13	0,12	0,12
6	0,13	0,12	0,16
7	0,47	0,60	0,49
9	0,13	0,09	0,12
10	0,23	0,12	0,05
11	0,17	0,08	0,05
12	0,37	0,09	0,22
13	0,40	0,33	0,46
14	0,10	0,16	0,16
15	0,17	0,08	0,17
Maximum	0,47	0,60	0,49
$\overline{\mathrm{x} }$	0,22	0,17	0,19

### Design

A first prototype, which has already been tested on patients, is made of grey PA12 and has a minimum wall thickness of 0.7 mm and a weight of 136 g.

Closure system: When selecting the closure system, it is of decisive importance that no small parts protrude which could hinder the child when lying down or pose a risk of injury. The closure should be quick and easy to operate—even when the child is moving. It should be possible to close the helmet with one hand by sliding the front part onto the back part. The back of the head can be fixed with the other hand. This makes the helmet easier to handle. In addition, there is no danger of the child being able to open the helmet by itself, as this would require pulling out the buckle in addition to pressing it together.

**Fig. 7:**
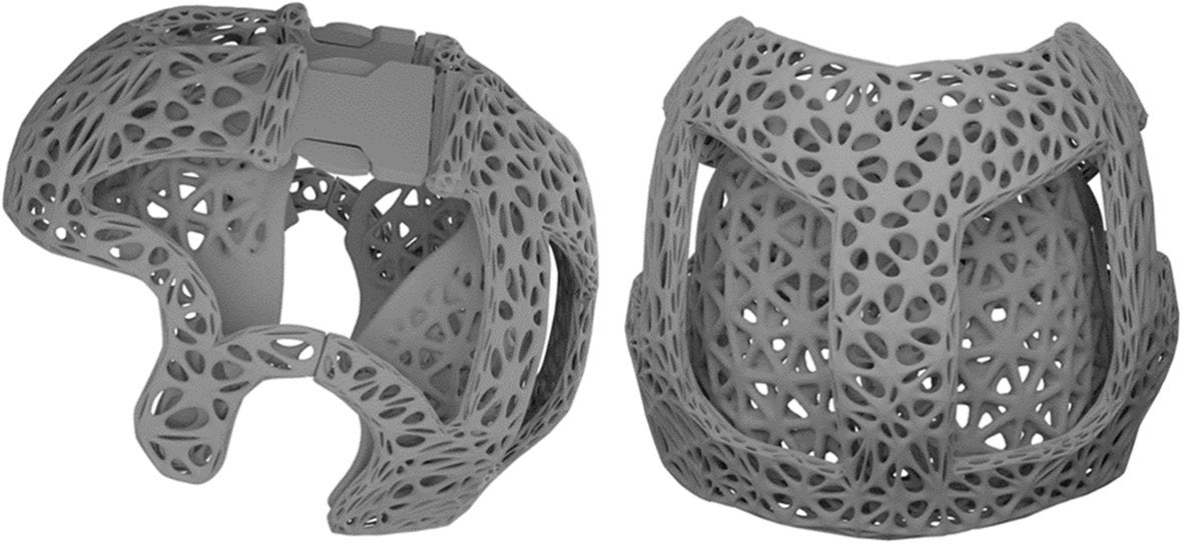
Prototype in grey PA12

**Fig. 8:**
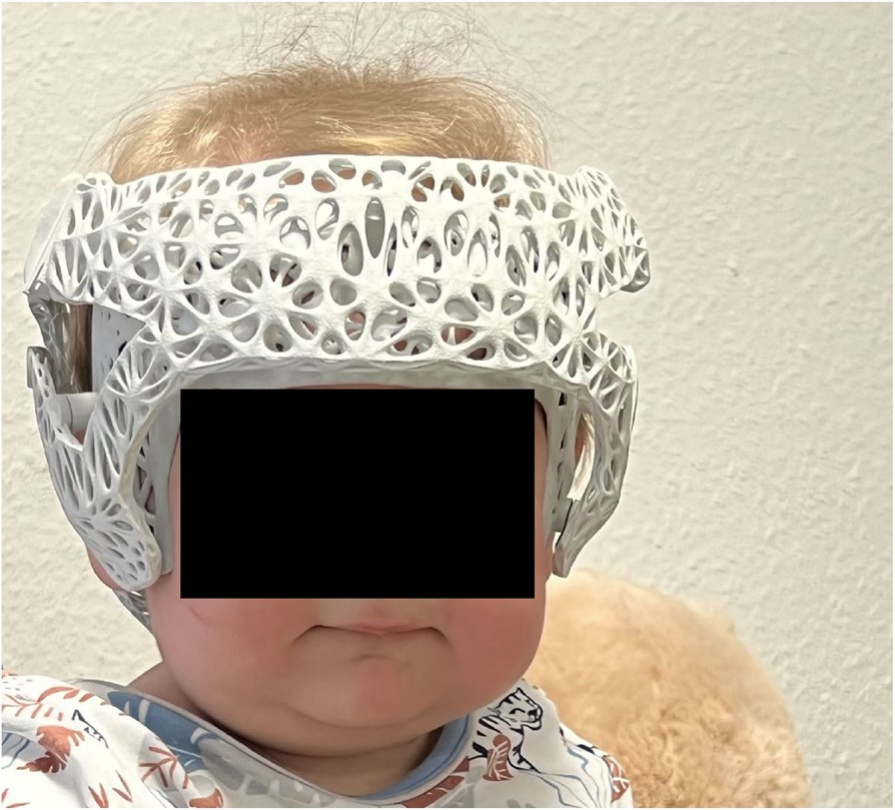
Fitting of the helmet orthosis with assessment of the accuracy of fit by a specialist orthopaedic technician

Other possible solutions included the use of a twist lock and a lever lock, which are already used in other types of orthoses. The final solution was the use of buckles, which is a tried and tested assembly technique. The buckles are inserted into each other, whereby the two outer fins hook into the counterpart and thus form a positive lock Figs. 7 and 8.

The final helmet concept consists of four individual helmet layers, two each forming an inner and outer helmet layer. The inner helmet layer is attached to the outer layer via the push-button system and can be replaced during the course of therapy. The outer helmet layer functions as the primary protective layer against external influences. The two parts of the outer layer are connected to each other via the push-in buckle system on the one hand and are simultaneously pre-adjusted via two guide bars.

The helmet is provided overall, all parts, with a Gtiterstrutkur. There were several design variants for this, with the wireframe variant offering the best combination of stability and ventilation. Lattice structures were also investigated.

### Manufacturing with 3D printing

The orthosis was 3D printed with Power Bed Fusion (HP MJF 580 Color printer, California, USA) of the research group, which is located at the university hospital. This printer is equipped with colour agents and thus enables colourful orthoses in individual designs.Polyamide 12 is a thermoplastic material commonly used in 3D printing. It offers good strength, stiffness, and durability, as well as an appealing surface structure. The printing process for the helmet can take up to 12 h, depending on the build chamber utilization. Following the printing process, the helmet needs to undergo depowdering to remove excess powder that was not melted or bonded during 3D printing. Depowdering can be accomplished using various methods such as brushing, suction, or air blasting. After depowdering, the helmet undergoes post-processing in a bead blasting chamber using glass beads. This step aims to smooth the surface of the helmet and eliminate any irregularities. The helmet is treated with a fine stream of abrasive particles in the blasting chamber to polish the surface, resulting in a uniform texture and a high-quality appearance. The post-processing in the blasting chamber can also enhance the adhesion of paints, coatings, or other surface treatments if desired. Once the helmet has completed the post-processing, it is ready for use and exhibits a beautiful surface structure achieved through the 3D printing process and post-treatment.

## Discussion

### 3D-Scan

The completed measurements were intended to show whether the scanning methodology used can enable a sufficient fit of the helmet orthosis and which parameters achieve further influences.

The maximum deviation between digital and analogue measurement is 4.7 mm. This deviation is within the range of the measurement deviation of 5 mm of the tape measure. The deviation could therefore also be due to the tape measure instead of the scanning methodology. Thus, the reliability of the measurement is limited, and thus the significance of the results is limited.

The average deviation between digital and analogue measured value was 2.2 mm, with large deviations of more than 3 mm being rather the exception.

With 0.1 mm accuracy according to the manufacturer's specification, the cause of the deviations lies more in the execution of the scanning process. The toddlers tend to "look behind" the scanner during the process and generally only rarely hold their heads extremely still. This is confirmed by the fact that the digital readings are generally larger than the analogue ones.

These values result in a high degree of inaccuracy regarding the fit, but a 5 mm thick Plastazote foam is used inside the helmet. This can adapt to the shape of the head and thus compensate for such inaccuracies. It should be noted, however, that the foam can also exert pressure on the head. The measurement method used is therefore sufficient, but it should be improved or modified in the future if possible.

### Manufacturing

The head orthosis was 3D printed to accommodate the individual design and complex shaping. material extrusion using fused depostion modeling was used for prototyping during the development process. Despite the lower costs of 3D printing with material extrusion, it is not an option for final production because of the mechanics of the final 3D printed parts as well as the required placement of support structures. Specfiically, the complex geometry has undercuts that can only be realised with support structures. However, this involves the risk of damage to the actual orthosis when the supports are removed, which could affect the strength.

Undercuts are particularly necessary when printing the buckle and push-button system. The intention in the design process was to design a functional system that can be used directly without time-consuming reworking and assembly.

Based on the accessibility of the 3D printers, the helmet concept could not be human tested on infants until the completion of this study. An application testing is crucial to validate the statements regarding the fit, effectiveness, compliance, and other improvements aimed for within the scope of this work.

Thus, the fit can only be evaluated through a helmet fitting and a longer duration of wear.

### Design

In the development process of the closure systems, the twist lock and the lever lock showed weaknesses, which led to the rejection of both systems. The design that used a buckle closure was preferred because of its low weight, ease of use, and low need for reworking. The buckles are sufficiently stable, durable and have no potentially dangerous geometries. A possible opening by the children is very unlikely.

**Fig. 9:**
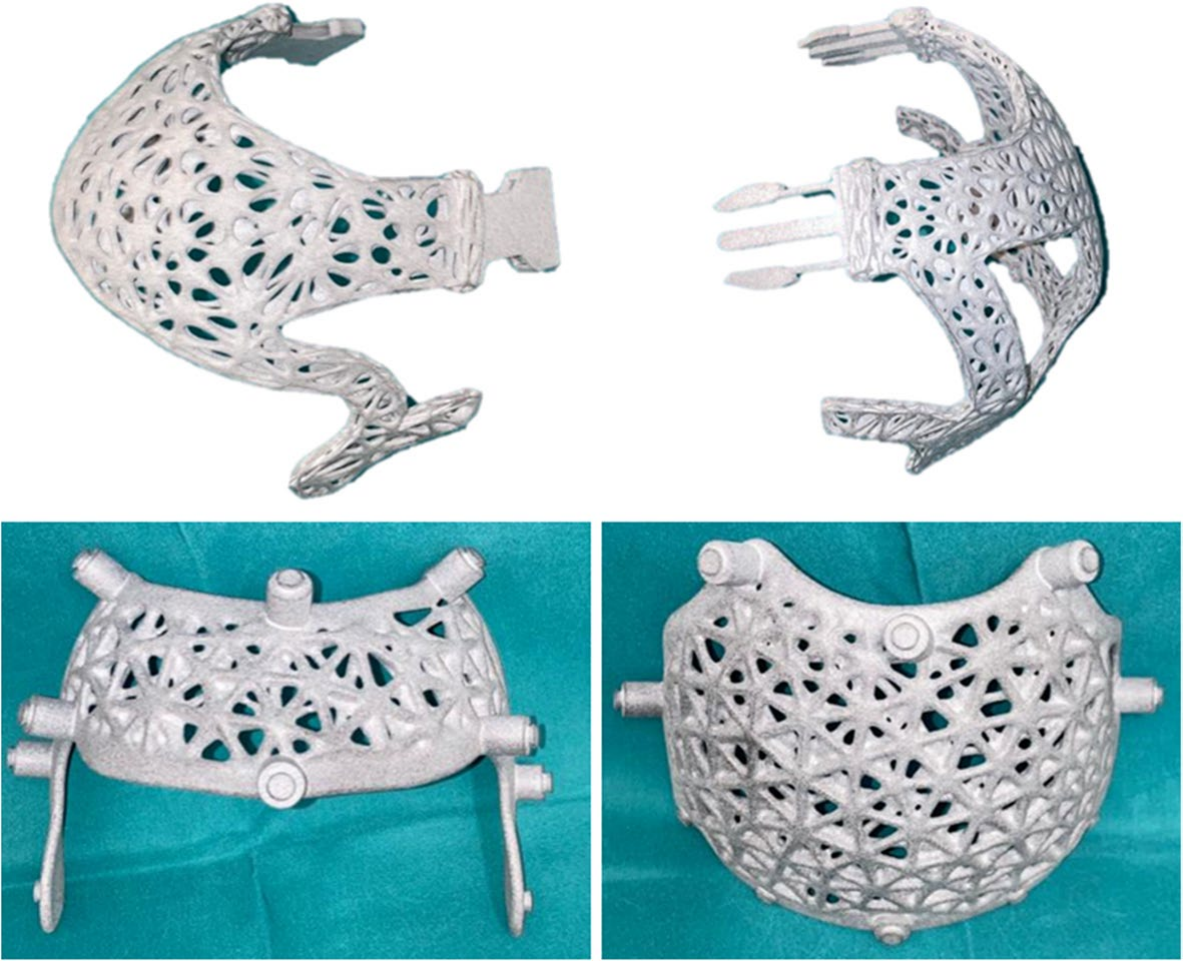
3D-printed final helmet concept consisting of PA12

The helmet orthosis can be customised by inserting ventilation holes. The improved aesthetics are expected to have a positive effect on compliance. The parents of the patients could help to design the orthosis, whereby a balance must always be struck between mechanical properties, i.e. stability, and the number of ventilation holes. The wireframe grid structure as illustrated (Fig. 9) proved to have sufficient mechanical stability despite the high number of holes. This makes large-area ventilation possible, which reduces sweating under the helmet.

The final helmet concept consists of a combination of a recessed outer layer, the wireframe grid structure, the push-button connection system and the push-in buckles.

Compared to conventional manufacturing methods for the helmet, the presented 3D-printed variant can be delivered more quickly. The current production process is based on a 3D scan for mold creation, which is outsourced. Subsequently, the helmet is customized using subtractive methods, which relies on the delivery times of the model manufacturer.

The costs do not significantly differ from conventional processes, as while material can be saved, PA12 is more expensive.

During the design phase of the helmet, several infants were utilized for 3D data acquisition. After production, the patients were scheduled for follow-up appointments, consistently yielding positive feedback regarding the design, ventilation, and innovation provided by the interchangeable inner part.

## Conclusion

If infants have plagiocephaly after permanent supine positioning, a specialist may indicate the need for a helmet orthosis. This aims to deflect the child's natural head growth towards a symmetrical shape. Since the helmet must be worn 23 h a day for several months to be successful, comfort, fit, weight and user-friendliness are decisive criteria for an effective helmet orthosis.

So far, the types of orthoses currently marketed have not found a solution to excessive sweating and associated itching and skin irritation. In addition, some systems run the risk of children opening them – either by intention or by accident. Therefore, 3D printed helmet orthoses based on a 3D scan are considered favorable and becoming to the fore. The rationale is that reliable closure concepts are directly integrated and inserted ventilation structures can significantly reduce sweating. Since, in contrast to conventional helmet orthoses, these cannot be ground to adapt to the child's growing head over the course of therapy, several concepts have already been developed to achieve this by other means. As these concepts struggle with a poor fit, the potential of 3D printing in the area of ventilation structures has not yet been fully exploited and the user-friendliness is limited, a new concept for an additively manufactured helmet orthosis was developed in this work.

A helmet concept that meets the requirements was developed with an innovative design and a secure closure system. In addition, a mobile 3D scanner that met the requirements was selected and the scanning methodology used was evaluated to guarantee the fit of the orthosis. 3D Printing with powder bed fusion served as a demand-oriented fabrication process so that the concept could be implemented and a prototype manufactured.

With the two-layer helmet system, the inner layer can be replaced during the course of therapy. Patients have to go for regular check-ups to check the fit of the helmet. Assuming that there are no defects in the solid component of the helmet (and therefore it does not need to be re-printed), the clinical follow-up will include a new 3D scan, from which a new inner layer is produced). This can be quickly and reliably fitted to the outer layer.

## Data Availability

The data are given in the paper and can be received on request.
